# Narsoplimab for severe transplant-associated thrombotic microangiopathy

**DOI:** 10.1186/s12959-023-00464-9

**Published:** 2023-03-13

**Authors:** Ambreen Pandrowala, Parth Ganatra, V. P. Krishnan, Ajay Narayan Sharma, Saroj Chavan, Minnie Bodhanwala, Bharat Agarwal, Prashant Hiwarkar

**Affiliations:** 1 Department of Blood and Marrow Transplantation, Bai Jerbai Wadia Hospital for Children, Acharya Donde Marg, Mumbai, 400012 India; 2 Department of Pediatric Radiology, Bai Jerbai Wadia Hospital for Children, Acharya Donde Marg, Mumbai, 400012 India; 3 Department of Pediatrics, Bai Jerbai Wadia Hospital for Children, Acharya Donde Marg, Mumbai, 400012 India; 4 Department of Pediatric Haematology-oncology, Bai Jerbai Wadia Hospital for Children, Acharya Donde Marg, Mumbai, 400012 India

**Keywords:** Transplant-associated thrombotic microangiopathy, Narsoplimab, Lectin pathway inhibitor

## Abstract

**Background:**

Transplantation-associated thrombotic microangiopathy (TA-TMA) is an endothelial injury syndrome linked to the overactivation of complement pathways. It manifests with microangiopathic hemolytic anemia, consumptive thrombocytopenia, and microvascular thrombosis leading to ischemic tissue injury. Mannose residues on fungi and viruses activate the mannose-binding lectin complement pathway, and hence activation of the lectin pathway could be one of the reasons for triggering TA-TMA. Narsoplimab, a human monoclonal antibody targeting MASP-2 is a potent inhibitor of the lectin pathway. We describe the transplant course of a pediatric patient who developed TA-TMA following Candida-triggered macrophage activation syndrome and was treated with Narsoplimab. The data collection was performed prospectively.

**Case presentation:**

The six-year-old girl underwent a human leucocyte antigen (HLA) haploidentical hematopoietic stem cell transplant using post-transplant Cyclophosphamide for severe aplastic anemia. In the second week of the transplant, the patient developed macrophage activation syndrome necessitating treatment with steroids and intravenous immunoglobulin. Subsequently, USG abdomen and blood fungal PCR revealed the diagnosis of hepatosplenic candidiasis. Candida-triggered macrophage activation syndrome responded to antifungals, steroids, intravenous immunoglobulin, and alemtuzumab. However, the subsequent clinical course was complicated by thrombotic microangiopathy. The patient developed hypertension in the 2nd week, followed by high lactate dehydrogenase (1010 U/L), schistocytes (5 per hpf), low haptoglobin (< 5 mg/dl), thrombocytopenia, and anemia in the 3rd week. Ciclosporin was stopped, and the patient was treated with 10 days of defibrotide without response. The course was further complicated by the involvement of the gastrointestinal tract and kidneys. She had per rectal bleeding with frequent but low-volume stools, severe abdominal pain, and hypoalbuminemia with a rising urine protein:creatinine ratio. Narsoplimab was started in the 5th week of the transplant. A fall in lactate dehydrogenase was observed after starting Narsoplimab. This was followed by the resolution of gastrointestinal symptoms, proteinuria, and recovery of cytopenia. The second episode of TA-TMA occurred with parvoviraemia and was also successfully treated with Narsoplimab.

**Conclusion:**

Lectin pathway inhibition could be useful in treating the fatal complication of transplant-associated thrombotic microangiopathy.

## Background

Complement activation caused by endothelial injury during stem cell transplant occurs due to toxicities of medicines used for conditioning, use of immunosuppressant medications, and infection [1]. Hence, blocking the terminal part of the complement system with Eculizumab has been tested with encouraging results [2]. The lectin pathway of the complement system is also activated by pathogens and plays an important role in microvascular endothelial cell injury. MASP-2 is the key enzyme responsible for activating and amplifying the lectin pathway [3]. MASP-2 deposition in the skin and bone marrow microvasculature has been reported in patients with TA-TMA [4]. Narsoplimab is a human immunoglobulin gamma4 monoclonal antibody targeting MASP-2. It blocks the lectin pathway effectively without affecting the activation of the classical pathway. We describe the use of Narsoplimab in a pediatric patient with severe aplastic anemia who developed severe TA-TMA after a haploidentical allogeneic hematopoietic stem cell transplant.

## Case report

A 6-year-old female child was diagnosed with acquired aplastic anemia in December 2019. Cytogenetic studies showed 46XX karyotype, chromosomal breakage study was normal, paroxysmal nocturnal hemoglobinuria clone was absent and clinical exome did not show any genetic mutation for inherited bone marrow failure syndrome. She did not have any significant infection during the pre-transplant course, except for the SARS-CoV-2 infection in June 2020. As she had no matched sibling or matched unrelated donor, she received a haploidentical stem cell transplant in July 2020.

The conditioning regimen was Rabbit ATG from Day-9 to Day-7 (0.5 mg/kg for 1 day and 2 mg/kg for 2 days), Fludarabine 40 mg/m^2^ for 4 days (Day-6 to Day-3), Cyclophosphamide 14.5 mg/kg for 2 days (Day − 6 and Day-5) and single fraction TBI (400 cGy) on Day − 1. On the day of transplantation, she received a peripheral blood stem cell graft from the father with 10 million CD34+ cells/kg and 2.41 × 10^8^ CD3+ T cells/kg. Post-transplant Cyclophosphamide (50 mg/kg) was given on Day+ 3 and Day+ 4 followed by Ciclosporin and Mycophenolate mofetil on Day+ 5 as graft-versus-host disease (GvHD) prophylaxis.

On Day+ 9, she developed high-grade fever with the onset of engraftment. Her ferritin was raised at 65000 ng/mL and her bone marrow had haemophagocytosis. Blood cultures were negative. On day+ 13, a diagnosis of *Candida tropicalis*-driven macrophage activation syndrome was established on blood PCR and radio imaging of the abdomen. She was treated with antifungals (caspofungin and posaconazole), intravenous immunoglobulin (1 g/kg for 5 days), and intravenous Methylprednisolone (2 mg/kg). She continued to have high-grade fever and ferritin peaked at 190,000 ng/ml on Day+ 13. Hence, one dose of intravenous Alemtuzumab (0.1 mg/kg) was administered following which her fever subsided.

The patient developed hypertension from Day+ 13 requiring three antihypertensive medications. On Day+ 14, she had loose motions with abdominal pain. Abdominal pain persisted with frequent loose motions and significant hematochezia. An ultrasound scan of the abdomen was suggestive of pan-colitis. Oral Budesonide was started for possible gut GvHD, and intravenous Methylprednisolone was continued considering the possibility of stage 4 gut GvHD. The patient had neutrophil engraftment on day+ 16 and the neutrophils continued to increase. However, hemoglobin and platelet count gradually decreased. TA-TMA was suspected because of anemia, thrombocytopenia, hypertension, gastrointestinal bleeding, decreased serum albumin, and raised lactate dehydrogenase. On Day+ 20, the blood smear showed 1 fragmented RBC per high power field, and on Day+ 37 fragmented RBCs peaked at 5 per high power field. Urine protein:creatinine ratio was 0.47 on Day+ 20, and the ratio peaked at 2.88 on Day+ 34. Serum haptoglobin level was very low (< 5 mg/dl), but the direct antiglobulin test was negative. Soluble C5b-9 assay is not available nationally and hence was not performed. ADAMTS-13 activity was normal. Based on the Overall-TMA criteria the diagnosis of TA-TMA was confirmed.

Ciclosporin was discontinued, and the patient was given Inj Defibrotide from Day+ 21. There was no evidence of pathogenic variation in TMA-associated genes on clinical exome. She continued to have laboratory and clinical parameters of TMA after 10 days of treatment with Defibrotide. Eculizumab, a terminal complement inhibitor was considered. Eculizumab is exorbitantly expensive and hence it was not possible to treat with Eculizumab. Narsoplimab was available through an expanded access program for compassionate use. Narsoplimab was therefore accessed and commenced at a dose of 4 mg/kg twice a week on Day+ 30.

The patient’s stool pattern changed from frequent small-volume stools to large-volume stools with ongoing hematochezia. Severe colitis with inflammatory thickening measuring 5.5 mm extending up to the caecum and base of the appendix was seen on an ultrasound scan. Since gut GvHD and TA-TMA are known to coexist, she was also started on a TNF-α inhibitor (Etanercept). She developed pneumatosis coli after two doses of Etanercept on Day+ 40 and Day+ 43, and hence Etanercept was stopped. She continued to have significant intestinal bleeding requiring 86 mls/kg of packed red cell transfusion for 7 days. Intestinal mucosa appeared friable on a serial ultrasound scan with a risk of perforation. Therefore, endoscopic biopsies were not attempted, and monitoring of intestines was done with serial ultrasonography.

**Fig. 1:**
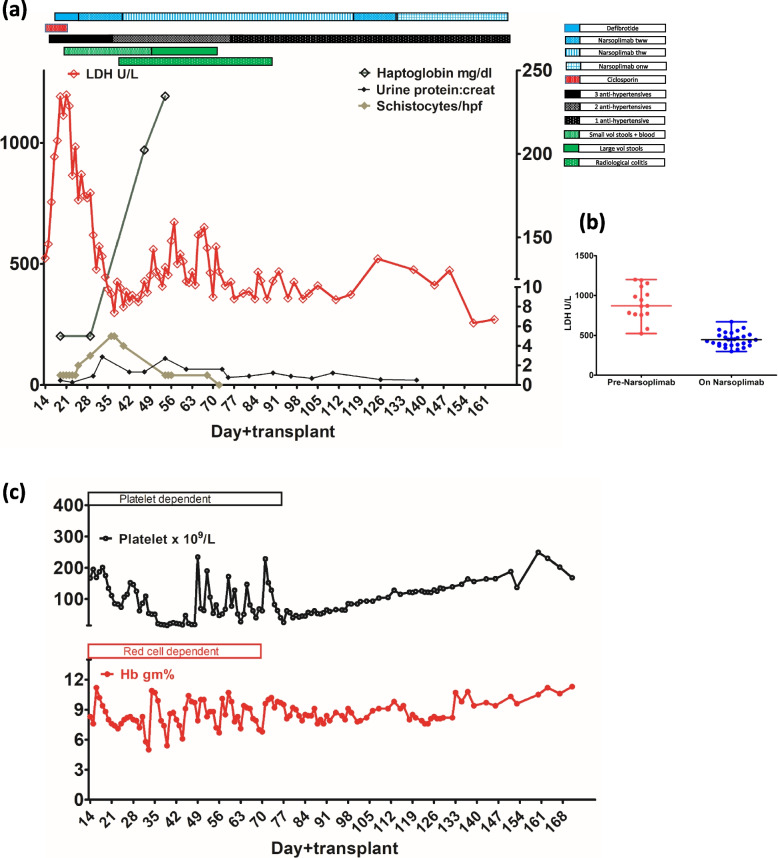
**a** Time course of clinical and laboratory markers of transplant-associated thrombotic microangiopathy is shown. Lactate dehydrogenase (LDH), haptoglobin, urine protein:creatinine ratio, schistocytes/hpf are plotted and events such as diarrhea, radiological colitis, and the number of anti-hypertensive medications and treatment of thrombotic microangiopathy with defibrotide and narsoplimab are shown. LDH is shown on the left y-axis and haptoglobin, urine protein:creatinine ratio, and schistocytes are shown on the right y-axis. **b** Lactate dehydrogenase (LDH) pre-narsoplimab and on treatment with narsoplimab are shown. A statistically significant difference in the LDH levels was observed on the unpaired t-test (*p* < 0.0001). **c** Hemoglobin and platelet trends during the early episode of TA-TMA are shown. The patient was red cell-dependent and platelet-dependent until day+ 70 and day+ 77 respectively. Abbreviations: ANC – absolute neutrophil count, LDH – lactate dehydrogenase, Hb – Hemoglobin, tww – twice a week, thw – thrice a week, onw – once a week, vol – volume

Narsoplimab was increased from twice a week to thrice a week on Day+ 45 because of significant intestinal bleeding. Her lactate dehydrogenase improved dramatically after starting Narsoplimab and her haptoglobin normalized on Day+ 48 (Fig. 1a). Her hypertension improved and her antihypertensive requirement was reduced to a single antihypertensive medication from Day+ 59 (Fig. 1a). Her intestinal bleeding reduced from Day+ 50 and completely stopped on Day+ 82. Inj Methyprednisolone was gradually tapered from Day+ 72 and stopped on Day+ 92 because of improvement in gut symptoms. There were no schistocytes in the peripheral blood smear after Day+ 70. The patient received the last packed cell transfusion on Day+ 70 and the last platelet transfusion on Day+ 77 (Fig. 1c). Narsoplimab was reduced to twice a week regimen on Day+ 109, a once-a-week regimen on Day+ 133, and stopped on Day+ 195 (Fig. 1a).

During this period, the patient had CMV reactivation on Day+ 59 with CMV copy numbers of 16,500/ml. She received Inj Foscarnet for 2 weeks, and CMV viremia resolved on Day+ 73. She had a herpes zoster skin infection on Day+ 111, which resolved with Acyclovir.

**Fig. 2:**
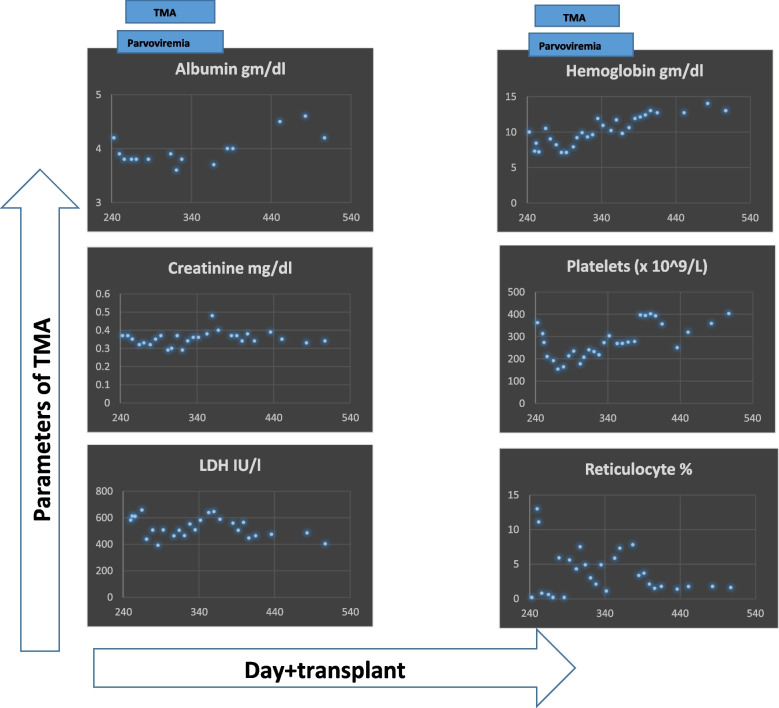
Second episode of TMA occurred concurrently with parvoviremia. Six parameters are shown: albumin, creatinine, LDH, hemoglobin, platelets, and reticulocyte percent. Some of these parameters, especially haematological ones, are affected in parvoviremia and TMA. However, albumin, creatinine, and LDH are primarily affected in TMA. Normalization of albumin, creatinine, and LDH marked the resolution of TMA

The patient developed liver GvHD from Day+ 217. Therefore, Tacrolimus was started from Day+ 217, and Ruxolitinib was added from Day+ 237. She had severe reticulocytopenia secondary to parvovirus infection on Day+ 246. Parvovirus infection was managed with immunoglobulin infusion. Concurrent with parvoviremia, she developed a second episode of TA-TMA manifested by circulating schistocytes, anemia, thrombocytopenia, low albumin, and an increase in lactate dehydrogenase and creatinine (Fig. 2). Urine protein:creatinine ratio increased to 0.89. Hence, Tacrolimus was stopped, steroids were started, and Ruxolitinib was continued. She was started on Narsoplimab from Day+ 254, initially twice a week for 3 weeks. Narsoplimab was escalated to thrice a week from Day+ 274 because of persistent TA-TMA, and then tapered to twice weekly from Day+ 290 and stopped on Day+ 357.

Liver GvHD resolved with steroids and Ruxolitinib. The patient is alive and well and had no features of TA-TMA on the last follow-up of D + 650.

## Discussion

The incidence of TA-TMA is as high as 35% in studies examining renal and other tissues [5]. HLA-mismatched transplant, acute GvHD, use of calcineurin inhibitor, more than one HSCT, and post-transplant invasive fungal infection are proven risk factors for developing TA-TMA [6, 7]. Our patient had multiple risk factors for TA-TMA such as a haploidentical transplant with Ciclosporin for GvHD prophylaxis, possible acute gut GvHD, and invasive fungal infection. In addition, our patient also had a covid-19 infection before the transplant. Covid-19 infection also causes endothelial damage and hence it is possible that having a covid-19 infection before the transplant may have contributed to TA-TMA [8].

A high index of suspicion and serial monitoring of laboratory parameters is required for the timely diagnosis of TA-TMA. Although renal biopsy can be unequivocally diagnostic, it is not practical for most patients. It is also noteworthy that gastrointestinal bleeding, diarrhea, and abdominal pain are features of both gut GvHD and intestinal TA-TMA. Continuous and refractory diarrhea with gastrointestinal bleeding in a patient who has received optimal treatment for gut GvHD suggests a possibility of co-existing intestinal TA-TMA [9]. Our patient was therefore treated with TMA-directed and GvHD-directed therapy. It is therefore not possible to be certain about the role of Narsoplimab in the resolution of gastrointestinal symptoms of our patient. However, there is a temporal association between the improvement in hematological parameters and the administration of Narsoplimab.

The treatment of TA-TMA with rituximab, plasmapheresis, and glucocorticoids similar to other microangiopathic diseases has not been effective. TA-TMA is endothelial dysfunction caused by complement dysregulation. Calcineurin inhibitors and acute GvHD cause endothelial damage and hence predispose to TA-TMA [6, 7]. Although we stopped her Ciclosporin, stopping a calcineurin inhibitor as a therapeutic intervention in a patient with a high risk of GvHD or active GvHD needs to be carefully considered. Defibrotide, an endothelial protector has also been used to treat TA-TMA with an approximately 55% response rate [10]. Defibrotide blocks the plasminogen activator inhibitor-1 and is the standard therapy for veno-occlusive disease, another transplant complication due to endothelial injury. It inhibits TNF-α mediated endothelial cell apoptosis as well as having anti-inflammatory, fibrinolytic and thrombolytic properties. More recently, targeting the complement system has emerged as a potential therapy with a better understanding of the pathophysiology of TA-TMA. Eculizumab is a humanized monoclonal antibody against complement protein C5. It inhibits the cleavage of C5 into C5a and C5b. Thus, Eculizumab acts in the terminal part of the complement cascade and prevents the formation of anaphylatoxin C5a. C5a has proinflammatory and prothrombotic properties, and it prevents the formation of membrane attack complex C5b-9, which results in endothelial cell death. Despite the encouraging response rates of 67-92% with Eculizumab, its use in the post-transplant setting is quite challenging [11]. Frequent monitoring of CH50 to suppress its value below 10% is required with Eculizumab [12]. Eculizumab blocks the effector arm of the immune response, making the patient vulnerable to encapsulated organisms such as meningococci and unable to mount an effective response to vaccines after transplant [13]. The cost of Eculizumab precludes its widespread use and hence additional therapies are required for the treatment of TA-TMA.

Narsoplimab, a lectin pathway inhibitor blocks the key effector enzyme MASP-2 without affecting the alternate pathway, and this may negate the risk of susceptibility to infections. We currently do not know the cost of Narsoplimab and there is no method available to assess lectin pathway inhibition. However, given the promising results in an adult phase 2 clinical trial [14] and the response seen in this case, clinical trials to further evaluate the safety and efficacy of lectin pathway inhibition in children are needed. In a phase 2 study of 28 adult patients with TA-TMA, the response rate was 61% [14]. One-hundred-day survival after HSCT-TMA diagnosis was 68% in the entire cohort and 94% in the responders. It was well tolerated without any significant toxicity in adults however there is no safety or efficacy data in children. This although a significant finding is not comparable with the previously published Eculizumab study because of differences in inclusion criteria [15, 16].

The prognosis of high-risk TA-TMA is dismal with a one-year survival rate of approximately 17% [17]. We report a pediatric patient who recovered from severe TA-TMA despite having multiple risk factors, such as very low haptoglobin, proteinuria > 30 mg/dl, and transplant with a haploidentical donor [18]. To our knowledge, this is the first case report of a pediatric patient treated with Narsoplimab. She tolerated Narsoplimab without any side effects. Early diagnosis and prompt treatment with targeted therapy, necessary supportive care, and aggressive management of triggering events can result in a cure for severe TA-TMA and the survival of the patient.

## Data Availability

Not applicable.

## References

[1] Chapin J, Shore T, Forsberg P, Desman G, Van Besien K, Laurence J (2014). Hematopoietic transplant-associated microangiopathy: case report and review of diagnosis and treatment. Clin Adv Hematol Oncol.

[2] Jodele S, Fukuda T, Vinks A, Mizuno K, Laskin BL, Goebel J (2013). Eculizumab therapy in children with severe hematopoietic stem cell transplantation-associated thrombotic microangiopathy. Biol Blood Marrow Transplant.

[3] Dodo J, Kocsis A, Gal P (2018). Be on target: strategies of targeting alternative and lectin pathway components in complement-mediated diseases. Front Immunol.

[4] Elhadad S, Chadburn A, Magro C, Van Besien K, Roberson EDO, Atkinson JP (2022). C5b-9 and MASP2 deposition in skin and bone marrow microvasculature characterize hematopoietic stem cell transplant-associated thrombotic microangiopathy. Bone Marrow Transplant.

[5] Laskin BL, Goebel J, Davies SM, Jodele S (2011). Small vessels, big trouble in the kidneys and beyond: hematopoietic stem cell transplantation-associated thrombotic microangiopathy. Blood..

[6] Elfeky R, Lucchini G, Lum SH, Ottaviano G, Builes N, Nademi Z (2020). New insights into risk factors for transplant-associated thrombotic microangiopathy in pediatric HSCT. Blood Adv.

[7] Harrison N, Mitterbauer M, Tobudic S, Kalhs P, Rabitsch W, Greinix H (2015). Incidence and characteristics of invasive fungal diseases in allogeneic hematopoietic stem cell transplant recipients: a retrospective cohort study. BMC Infect Dis.

[8] Niederwieser C, Weber B, Reichard M, Gagelmann N, Ajib S, Schlipfenbacher V (2022). Endothelial complications after allogeneic stem cell transplantation in patients with pretransplant resolved COVID-19. Bone Marrow Transplant.

[9] El-Bietar J, Warren M, Dandoy C, Myers KC, Lane A, Wallace G (2015). Histologic features of intestinal thrombotic microangiopathy in pediatric and young adult patients after hematopoietic stem cell transplantation. Biol Blood Marrow Transplant.

[10] Corti P, Uderzo C, Tagliabue A, Della Volpe A, Annaloro C, Tagliaferri E (2002). Defibrotide as a promising treatment for thrombotic thrombocytopenic purpura in patients undergoing bone marrow transplantation. Bone Marrow Transplant.

[11] Jodele S, Fukuda T, Vinks A, Mizuno K, Laskin BL, Goebel J (2014). Eculizumab therapy in children with severe hematopoietic stem cell transplantation associated thrombotic microangiopathy. Biol Blood Marrow Transplant.

[12] Jodele S, Fukuda T, Mizuno K, Vinks AA, Laskin BL, Goebel J (2016). Variable eculizumab clearance requires pharmacodynamic monitoring to optimize therapy for thrombotic microangiopathy after hematopoietic stem cell transplantation. Biol Blood Marrow Transplant.

[13] Gäckler A, Kaulfuß M, Rohn H, Vogel U, Claus H, Feldkamp T (2020). Failure of first meningococcal vaccination in patients with atypical hemolytic uraemic syndrome treated with eculizumab. Nephrol Dial Transplant.

[14] Khaled SK, Claes K, Goh YT, Kwong YL, Leung N, Mendrek W (2022). OMS721-TMA-001 study group members. Narsoplimab, a Mannan-binding lectin-associated serine Protease-2 inhibitor, for the treatment of adult hematopoietic stem-cell transplantation-associated thrombotic Microangiopathy. J Clin Oncol.

[15] Jodele S, Dandoy CE, Lane A, Laskin BL, Teusink-Cross A, Myers KC, Wallace G, Nelson A, Bleesing J, Chima RS, Hirsch R, Ryan TD, Benoit S, Mizuno K, Warren M, Davies SM (2020). Complement blockade for TA-TMA: lessons learned from a large pediatric cohort treated with eculizumab. Blood..

[16] Zhang R, Zhou M, Qi J, Miao W, Zhang Z, Wu D, Han Y (2021). Efficacy and safety of Eculizumab in the treatment of transplant-associated thrombotic Microangiopathy: a systematic review and Meta-analysis. Front Immunol.

[17] Jodele S, Davies SM, Lane A, Khoury J, Dandoy C, Goebel J, Myers K, Grimley M, Bleesing J, El-Bietar J, Wallace G, Chima RS, Paff Z, Laskin BL (2014). Diagnostic and risk criteria for HSCT-associated thrombotic microangiopathy: a study in children and young adults. Blood..

[18] Schoettler M, Lehmann LE, Margossian S, Lee M, Kean LS, Kao PC (2020). Risk factors for transplant-associated thrombotic microangiopathy and mortality in a pediatric cohort. Blood Adv.

